# Effect of Employee–Customer Interaction Quality on Customers’ Prohibitive Voice Behaviors: Mediating Roles of Customer Trust and Identification

**DOI:** 10.3389/fpsyg.2021.773354

**Published:** 2021-12-14

**Authors:** Guofu Chen, Shuhao Li

**Affiliations:** School of Management, Xiamen University, Xiamen, China

**Keywords:** customer trust, customer identification, customer voice behaviors, employee–customer interaction quality, prohibitive voice behaviors, restaurant

## Abstract

Given that customer voice behaviors are confused with customer complaint behaviors in usage, this study thoroughly explains the essential differences between the two constructs. On that basis, this study investigates how employee–customer interaction (ECI) quality affects customers’ prohibitive voice behaviors, which is an crucial type of customer voice behaviors, by examining customer trust and identification as mediators. Data from 395 restaurant customers are collected and analyzed using structural equation modeling. Results show that ECI quality positively affects customers’ prohibitive voice behaviors. In this effect, customer trust and identification play direct and sequential mediating roles. This study contributes theoretically to the current knowledge by clearly distinguishing customer voice behaviors from customer complaint behaviors and by providing new insights into the mechanism of customers’ prohibitive voice behaviors from the perspectives of service interaction and relational benefit enhancement. The practical implications of this study can help pointedly foster customers’ prohibitive voice behaviors.

## Introduction

Based on the service-dominant logic, customers are co-producers of value for enterprises (Vargo and Lusch, 2001; Fellesson and Salomonson, 2016; Dellaert, 2019). Thus, the notion of value co-creation has received increasing attention in marketing study and practice. Value co-creation addresses the roles of customers as “partial employees” who play joint roles with enterprises in creating value in the service production (Grissemann and Stokburger-Sauer, 2012; Halbesleben and Stoutner, 2013). Under the concept of value co-creation, the communication modes between enterprises and customers have changed from one-way delivery to mutual dialog, which implies that enterprises can obtain customer feedback to gain helpful information and resources for improvement (Payne et al., 2008; Zhao et al., 2019). Against this background, customer voice behaviors, conceptualized as customers’ extra-role communicative behaviors of proactively expressing their suggestions or opinions to enterprises have received considerable attention to strengthen customer dialogs with enterprises (Ran and Zhou, 2020). Customer voice behaviors present a unique opportunity for enterprises to receive customer feedback, which in turn provides much value for co-creation between customers and enterprises (Bai et al., 2020). Hence, enhancing customer voice behaviors in the era of value co-creation is considerably important.

Customer voice is an important manifestation of customers’ commitment to the company (Min and Kim, 2019; He et al., 2020). Unfortunately, existing study confused customers’ voice and complaint behaviors (Wei et al., 2012; Raval, 2020). Many scholars analyzed the relationship between consumer complaint behaviors and loyalty and satisfaction (Raval, 2020). Research on consumer voice behaviors currently pays much attention to the effect of employee voice on corporate performance (Kaufman, 2014; Lin and Johnson, 2014; Chou et al., 2019; Torre et al., 2021). Besides, extant research explored the influencing factors of customer voice behaviors including satisfaction, loyalty and right (Yoon and Kim, 2016; Briñol et al., 2017; Rather and Sharma, 2017; Wan and Li, 2021). These studies mainly focused on the interactive relationship between customers and enterprises, while ignoring the interactive relationship between customers and employees. In addition, existing scholars mainly analyzed customer engagement and loyalty from customer brand identification (So et al., 2013; Rather et al., 2019; Raza et al., 2020), ignoring the mediating role of customer trust and identification in the relationship between employee–customer interaction and customer’s voice behaviors. Customer trust is an important construct in relationship marketing theory, which deals with customers’ confident beliefs about the integrity and reliability of an enterprise (Jose, 2013). Customer identification is demonstrated as positively related to customer satisfaction, loyalty, purchase intention, and product utilization behaviors (Li et al., 2021). The mediating role of customer identification between external stimuli and customer behavioral responses has been verified (Paulssen et al., 2019; Yang and Hao, 2020). Therefore, this study explores the mediating role of customer trust and identification in customer-employee interaction quality and customer voice behaviors.

The underlying mechanism of customer voice behaviors are increasingly investigated by highlighting the roles of customer inclusion, identification, and the perceived trustworthiness and power of service workers (Bove and Robertson, 2005; Béal and Sabadie, 2018; Ran and Zhou, 2019). However, existing studies have four clear drawbacks. First, customer voice behaviors are considerably confused with customer complaint behaviors. Boote (1998) refers to customer voice as an act of complaint, which expresses complaints verbally to service providers (Marquis and Filiatrault, 2002; Bove and Robertson, 2005). However, this definition of customer voice behaviors has been unable to meet the current actual situation and research. Assaf et al. (2015) proposed that customer satisfaction and complaints are two basic customer voice variables, while Béal and Sabadie (2018) divided customer voice behaviors into two dimensions, such as complaint intention and service improvement suggestions. In those studies, complaints and suggestions are all customer voices (Cossío-Silva et al., 2016; Béal and Sabadie, 2018). In general, the two terms are mixed in usage (Boote, 1998; Béal and Sabadie, 2018; Ran and Zhou, 2019), which casts a negative light on the understanding of the underlying mechanisms of these two behaviors. Therefore, the essential differences of the two constructs require clear explanation.

Second, little importance is paid to the roles of employees in the formation of customer voice behaviors, despite their inevitable customer interactions in the consumption process (Huang and Xie, 2017). Indeed, the influences of employee–customer interaction (ECI) quality on customer evaluations of service encounters and post-purchase behaviors are well documented (Zolfagharian et al., 2018; Wang and Lang, 2019). Unfortunately, the effect of ECI quality on customer voice behaviors is rarely explored. Bove and Robertson (2005) proposed that trust is a predicting factor of customer voice behaviors. Yang and Hao (2020) found that customer identification affects promotive voice behavior. Although these scholars explored the influencing factors of voice behaviors, but these belong to proximal factors. And the distal factors and mechanisms of customer voice behaviors need to be further explored. In addition, although the relationship between the enterprise and the customer will affect the customer voice behaviors. However, the relationship between ECI quality and customer voice behaviors is full of uncertainty, especially customers’ prohibitive voice behaviors.

Third, customer voice behaviors include promotive and prohibitive aspects (Ran and Zhou, 2020), but existing research pays great importance to the former (Chamberlin et al., 2017; Wang and Lang, 2019; Hudson et al., 2020; Yu et al., 2020) while fairly neglecting the latter. Béal and Sabadie (2018) found that psychological ownership stimulates customer voice behaviors. The underlying mechanism of customers’ prohibitive voice behaviors are expected to be further explored. Fourth, the role of customer identification in driving customer voice behaviors is examined (Ran and Zhou, 2019, 2020; Rather et al., 2019; Yang and Hao, 2020), but the factors that drive customer identification are not considered. Thus, limited managerial implications are provided for the promotion of customer voice behaviors. For instance, Rather et al. (2019) indicated that customer brand identification, affective commitment, customer satisfaction, and brand trust as antecedents of customer behavioral intention of loyalty. Thus, limited managerial implications are provided for the promotion of customer voice behaviors. Although these scholars explore the impact of customer brand identification on loyalty behavior and organizational citizenship behavior, these are still near-end factors, and it is necessary to explore more remote influencing factors and mechanisms of customer voice behaviors.

To fill the gaps, the present study investigates the effect of ECI quality on customers’ prohibitive voice behaviors by examining the mediating roles of customer trust and identification on the basis of clear distinctions between customer voice and complaint behaviors. The significant contributions of this study are as follows. First, the essential differences between customer voice and complaint behaviors are thoroughly explained, which provides a solid foundation for the understanding of the underlying mechanism of these two behaviors. Drawing on the study of Chamberlin et al. (2017) and Yu et al. (2020) on the classification of employee voices, we classify customer voices into promotive and prohibitive voices, and address the gap in customer voice behavior. Second, existing research explores the impact of customer corporate identity (Yang and Hao, 2020) on customer’s voice behavior, while this article explores more remote factors and verifies the positive behavior of customers on service contact and after purchase by Zolfagharian et al. (2018). This paper finds that ECI quality has a positive impact on customers’ prohibitive voice behaviors. This study presents a pioneer attempt to explore the role of ECI quality in predicting customers’ prohibitive voice behaviors. This study is a valuable addition to the antecedents of such behaviors from the perspective of service interactions and provides helpful practical implications for its stimulation.

Third, the findings of this paper extend the research of Wang and Lang (2019) and find that the quality of customer-employee interaction can improve customers’ prohibitive voice behaviors through customer identification, not just promotive voices. This study supports the positive effects of Grissemann and Stokburger-Sauer (2012) and Ran and Zhou (2020) on the role of customers as “partial employees.” In addition, the results of this research show that customer trust and identification have a positive impact on customers’ prohibitive voice behaviors. This validates the work of Roy et al. (2018) and Ran and Zhou (2019, 2020) and expands its application areas. Finally, two relational variables (i.e., customer trust and identification) are integrated to open the “black boxes” of how ECI quality indirectly affects customers’ prohibitive voice behaviors by building relational benefits. This integration considerably improves the works of Ran and Zhou (2019, 2020) that pay limited attention to the upstream predictors of customer identification. This paper finds that customer trust and identification play sequential mediating roles ECI quality on customers’ prohibitive voice behaviors. This result further rationalizes the logical relationship between employee-customer relationship quality, customer trust, customer identification and customers’ voice behaviors.

## Literature Review and Research Hypothesis

### Customer Voice Behaviors

The concept of customer voice behaviors extends from that of employees in organizational behavior research (Ran and Zhou, 2019, 2020). Dyne and Lepine (1998) argue that employee voice behaviors act as extra-role communicative behaviors in which employees initiate putting forward constructive suggestions and opinions to organizations. Similarly, customer voice behaviors refer to customers’ proactively vocal expressions of providing feedback or viewpoints to enterprises beyond their required roles (Ran and Zhou, 2020). In light of the opinions of Groth (2005) and Yi and Gong (2006), customer voice behaviors describe that customer provide useful feedback information, particularly suggestions and opinions for product and service improvement to enterprises. Yang and Hao (2020) defined customer voice behavior as the role of consumers providing suggestions or opinions to the enterprise. Essentially, this feedback provision pertains to customer citizenship behaviors. Surprisingly, numerous studies regard customer complaint behaviors, which obviously do not pertain to customer citizenship, as a type of customer voice behaviors (Assaf et al., 2015; Béal and Sabadie, 2018; Won-Moo et al., 2018; Ran and Zhou, 2019), which is a considerable misinterpretation. Thus, distinguishing these two behaviors is necessary before conducting empirical research.

Based on the well-documented distinctions between employee voice and complaint behaviors (Barry and Wilkinson, 2016; Lu and Liu, 2016; Nechanska et al., 2020), the present study argues that customer voice and complaint behaviors have essential differences, and that the latter does not pertain to the former. Specifically, the basic purpose of customer voice behaviors is to improve enterprise performance by actively expressing customer suggestions and opinions (Ran and Zhou, 2020), while that of customer complaint behaviors is to express customer discontent, vent negative emotions, and even seek compensation from enterprises (Day et al., 1981; Bitner et al., 1990; Yang and Hao, 2020). In addition, customer complaint behaviors generally occur on the premise of dissatisfaction, that is, customers generally complain to employees when encountering unpleasant experiences in the consumption (Day et al., 1981; Bitner et al., 1990). Meanwhile, customer voice behaviors can develop in the absence of displeasure, that is, customers are likely to exhibit vocal suggestions and opinions to enterprises when they do not encounter dissatisfaction (Ran and Zhou, 2020). Essentially, customer voice behaviors pertain to a type of customer citizenship behaviors (Groth, 2005; Yi and Gong, 2006; Ran and Zhou, 2020), whereas customer complaint behaviors do not. Therefore, the influences of customer voice behaviors on enterprises are generally positive whereas those of customer complaint behaviors tend to be negative.

According to the above arguments and in accordance with the opinions of Ran and Zhou (2020), the present study divides customer voice behaviors into two types: customers’ promotive and prohibitive voice behaviors. The former refers to customer expressions of innovative suggestions and ideas for improvement of products and services of enterprises, while the latter refers to customer proactive behaviors of kindly informing enterprises of practical and potential problems that are not conducive to enterprise development (Ran and Zhou, 2020). The underlying mechanism of customer voice behaviors has been investigated (Bove and Robertson, 2005; Béal and Sabadie, 2018; Ran and Zhou, 2019, 2020). However, these studies mainly focus on the drivers of customer voice behaviors regarding its promotive aspect, but pay limited attention to the prohibitive dimension (Béal and Sabadie, 2018; Ran and Zhou, 2019) despite the latter’s contribution to enterprises by preventing potential problems and correcting actual errors (Ran and Zhou, 2020). The role of ECI quality in predicting customer voice behaviors is also scarcely examined despite its important influence on customer attitudes and behaviors (Zolfagharian et al., 2018; Wang and Lang, 2019). Accordingly, the present study highlights customers’ prohibitive voice behaviors and empirically examines the effect of ECI quality on such behaviors.

### Effect of Employee–Customer Interaction Quality on Customer Prohibitive Voice Behaviors

Employee–customer interaction (ECI) is a dynamic process that generally runs through the consumption experience within the context of high-contact service delivery, such as restaurants and hotels (Huang and Xie, 2017). From the customer perspective, ECI quality is defined as customer perceptions of the superiority of their interactions with employees. High-quality ECI are characterized as customers’ sense of effectiveness, helpfulness, and comfort regarding their interactions with employees (Lin and Wong, 2020). Customers generally deal with employees when receiving services in a hospitality setting. Thus, the roles of employees in improving customer experience must not be underestimated. Employee–customer interaction quality is conductive to improving customer–employee connections and customer satisfaction and loyalty (So et al., 2013; Ranjan et al., 2015; Kaminakis et al., 2019; Wang and Lang, 2019), and moderates the positive effect of customer–to–customer interactions on brand experience (Lin and Wong, 2020). Nonetheless, scant research empirically explores the effect of ECI quality on customers’ extra-role behaviors (customers’ prohibitive voice behaviors). Therefore, the present study is carried out to fill this gap.

This study proposes that ECI quality is positively associated with customers’ prohibitive voice behaviors. High-quality ECI is characterized as customers receiving personal care, useful help, and sense of genuineness from employees. The customer–employee interaction can be regarded as a type of social exchange relationship. According to the reciprocity principle in social exchange theory (Blau, 1964), if customers sense a gain of personal benefits in high-quality ECI from employees of enterprises, then they are inclined to do or give a positive act for the employees or the enterprises as rewards in the social exchange relationship. Customers’ prohibitive voice behaviors essentially pertain to a type of customer citizenship behaviors (Groth, 2005; Ran and Zhou, 2020), which can be viewed as customers’ positive rewards for employees or enterprises. Hence, in light of social exchange theory, this study argues that ECI quality positively affects customers’ prohibitive voice behaviors. In addition, Yi and Gong (2006) also reveal that, as important manifestations of high-quality ECI, perceived personal attention and courteousness from employees are positively associated with customer citizenship behaviors. Therefore, this study proposes that ECI quality is positively associated with customers’ prohibitive voice behaviors. Accordingly, the present study proposes that:

H1. ECI quality positively influences customers’ prohibitive voice behaviors.

### Direct Mediating Role of Customer Trust

Customer trust is an important construct in relationship marketing theory, which deals with customers’ confident beliefs about the integrity and reliability of an enterprise (Morgan and Hunt, 1994). If customers perceive that an enterprise performs as promised or expected, then the former are inclined to believe that the latter is trustworthy and has high credibility (Kim et al., 2018). Morgan and Hunt (1994) argue that trust occurs when individuals have faith in an exchange partner’s credibility and integrity, and customer trust is the antecedent of customer commitment (Morgan and Hunt, 1994; Martínez and Bosque, 2013). Accordingly, gaining customer trust is considered the prerequisite for customer loyalty (Reichheld and Schefter, 2000; Martínez and Bosque, 2013; Ranganathan et al., 2013).

High-quality ECI marked by promptness, courtesy, and thoughtfulness is regarded as a service provider–customer interface (Hartline and Ferrell, 1996; Lee et al., 2014). Service is intangible and inseparable, and therefore customers generally evaluate service enterprises on the basis of their perceptions of how well employees treat them. This view supports that higher ECI quality leads to the higher likelihood that customers perceive the organization as reliable and trustworthy (Yieh et al., 2007; Lee et al., 2014). Moreover, rapport is characterized by enjoyable ECI and helps decrease uncertainty and increase confidence (Macintosh, 2009). If customers gain personal care and attention from employees in service interactions, then they tend to develop favorable evaluations toward employees and thereby establish trust-based relationships with service enterprises (Matute et al., 2018). In addition, many existing studies provide statistical evidence for the role of ECI quality in driving customer trust (Yieh et al., 2007; Lee et al., 2014). Employee–customer interaction quality generate emotional trust of customer (Johnson and Grayson, 2005; Kumar et al., 2013). Hence, this study suggests that:

H2. ECI quality positively influences customer trust.

Based on social psychology literature, customer trust is divided into cognitive and emotional trust (Mcallister, 1995; Dirks and Ferrin, 2002; Punyatoya, 2019). Cognitive trust is based on the values, experience, and information cues shared between customers and service providers, which is the customer’s confidence or willingness to rely on the service provider (Johnson and Grayson, 2005; Castaldo, 2007; Sekhon et al., 2013). Emotional trust is the result of ECI. If service providers give more care to customers, deep emotions can be established between them (Johnson and Grayson, 2005; Harms et al., 2016). At this time, customers are more willing to make voices behaviors. Therefore, customer trust promotes customers’ voices behaviors.

A high level of trust allows customers to gain much confidence in service providers, and drives them to act as advocators for enterprises (Gremler et al., 2001). Undoubtedly, advocating customers are inclined to voluntarily exhibit voice behaviors toward enterprises for its long-term development. On this basis, Morgan and Hunt (1994) early point out that customer trust can lead to customer cooperation behaviors, which are a type of customers’ voluntary behaviors. According to social exchange theory, customer trust is a key component of relationship utility, which can enhance customers’ expectations of long-term mutual benefit. Therefore, customer trust is expected to promote customers’ supportive extra-role behaviors toward enterprises on the basis of the reciprocity principle suggested by social exchange theory (Blau, 1964). Customers are “partial employees” of the company, and their trust and sense of responsibility in the company will promote their voice behaviors (Fuller et al., 2006; Chamberlin et al., 2017; Arain et al., 2019). Furthermore, past evidence supports the positive effect of trust on customers’ beneficial behaviors toward enterprises, such as raising helpful ideas for improvements and providing feedback on service-related problems (Di et al., 2010; Kim et al., 2018; Roy et al., 2018). In essence, customers’ prohibitive voice behaviors are customers’ supportive and beneficial behaviors beyond their required roles (Ran and Zhou, 2019, 2020). Thus, this study puts forward that:

H3. Customer trust positively influences customers’ prohibitive voice behaviors.

Numerous studies confirm the mediating role of customer trust in the linkages between external driving factors and customer behaviors (Di et al., 2010; Martínez and Bosque, 2013; Kim et al., 2018). Apiradee and Nuttapol (2018) found online sellers can build customer engagement with customer trust as mediators. Yu et al. (2020) explored the influence of corporate social responsibility image on the consumption behavior and co-developing behavior of customers. Chen et al. (2021) found that cognitive trust increases people’s willingness to disclose information and reduces their willingness to falsify it. Delgosha and Hajiheydari (2021) drawing on trust in technology model, proposes a conceptual dual model to explain robot users’ post-adoption behaviors, while considers the mediating roles of trust. In light of relationship marketing theory, customer trust serves as a mediator to link stimulating factors and customer behavioral responses (Morgan and Hunt, 1994; Martínez and Bosque, 2013). Hence, the present study considers that ECI quality, as a type of social environmental stimulus, can help develop trust-based relationships between enterprises and customers, thereby indirectly fostering customers’ prohibitive voice behaviors toward enterprises. In other words, high-quality ECI is conductive to increasing customers’ perceptions of enterprise reliability and credibility, and such perceptions in turn drive customers to exhibit various voice behaviors toward enterprises. Therefore, this study hypothesizes that:

H4. Customer trust plays a direct mediating role in the positive effect of ECI quality on customers’ prohibitive voice behaviors.

### Direct Mediating Role of Customer Identification

Customer identification is defined as customer’ cognitive state of self-categorization, connection, and sense of belonging toward an enterprise (Bhattacharya and Sen, 2003). According to social identity theory, individuals have needs to develop social identity and self-definition; therefore, customers are inclined to build close relationships with enterprises by positioning themselves as part of the organization to satisfy their intrinsic motivations (Ahearne et al., 2005; Martínez and Bosque, 2013; Mohan et al., 2021). As the level of customer identification increases, the higher the likelihood that customers exhibit positive attitudes and behaviors toward an enterprise (Ahearne et al., 2005; Ran and Zhou, 2019). On this basis, customer identification is demonstrated as positively related to customer satisfaction, loyalty, purchase intention, and product utilization behaviors (Ahearne et al., 2005; Keh and Xie, 2009; Martínez and Bosque, 2013).

High-quality ECI is conductive to creating favorable associations between employees and customers, thereby providing a unique opportunity to establish positive customer–enterprise relationships (Yim et al., 2008). Customer identification is exactly an important form of such relationships. Hence, ECI quality is considered to foster customer identification. Enjoyable ECI is the basic element of customer experience of rapport, which occurs when customers have positive interactions with employees (Gremler et al., 2001). Linzmajer et al. (2020) suggest that rapport is an important antecedent of customer identification. Accordingly, the present study argues that ECI quality can predict customer identification. Moreover, Marín and Salvador (2013) suggest that ECI quality can increase customer identification with enterprises. Similarly, Homburg et al. (2009) indicate that enjoyable ECI helps develop customers’ social identity with enterprises. Therefore, the present study posits that:

H5. ECI quality positively influences customer identification.

In light of social identity theory, strong identification prompts individuals to regard themselves to have close connections with an organization, thus motivating them to act supportively toward the organization as if doing the same for their own sake (Ran and Zhou, 2020). Moreover, existing literature has empirically addressed the role of customer identification in promoting customers’ extra-role behaviors (Ahearne et al., 2005; Wu et al., 2017; Hur et al., 2018). Customer voice behaviors pertain to a type of customers’ extra-role behavior that helps organizations understand their shortcomings and improvement directions (Ran and Zhou, 2019, 2020). Therefore, this study argues that customer identification likely affects customer voice behaviors. Using 394 students as samples, Ran and Zhou (2020) also empirically examine the role of customer identification in predicting customers’ prohibitive and prohibitive voice behaviors. Wang and Lang (2019) pointed out that the customer identification will increase customers’ emotion and commitment to the brand. Yang and Hao (2020) found that customer identification is positively correlated with customers’ voices. Thus, the present study predicts that:

H6. Customer identification positively influences customers’ prohibitive voice behaviors.

The mediating role of customer identification in the relationship between external stimuli and customer behavioral responses has been extensively verified (Ahearne et al., 2005; Martínez and Bosque, 2013; Hur et al., 2018). Social identity theory also supports that customer identification is often applied to link enterprise-related factors and customer responses (Bhattacharya and Sen, 2003). Bove and Robertson (2005) proposed that relational variables will be an important antecedent of customer voices. Bhattacharya and Sen (2003) believe that customer identification can satisfy consumers’ psychological needs for self-identification, and then influence their behavior. Albert et al. (2020) think that customers identification will motivate to show voluntary and positive behavior toward the company. Existing research supports the view that consumer-enterprise identity has a positive effect on consumers’ extra-role behavior (Karaosmanoglu et al., 2011; Wu et al., 2017; Hur et al., 2018).

On this basis, the present study argues that customer identification may act as a mediator in the relationship between ECI quality and customers’ prohibitive voice behaviors. Specifically, as a crucial enterprise-related factor, ECI quality can potentially influence customers’ prohibitive voice behaviors through the indirect path of customer identification. In other words, ECI quality can indirectly stimulate customers’ prohibitive voice behaviors by promoting customer identification. Accordingly, this study proposes that:

H7. Customer identification plays a direct mediating role in the positive effect of ECI quality on customers’ prohibitive voice behaviors.

### Sequential Mediating Roles of Customer Trust and Identification

Previous research extensively supports the positive effect of customer trust on customer identification (Keh and Xie, 2009; Martínez and Bosque, 2013; Martíner and Ignacio, 2015). Customers generally have a good evaluation of enterprises they trust, on which basis they easily develop identification. Keh and Xie (2009) suggest that customers tend to identify with reliable and honest enterprises to facilitate self-definition and enhance self-esteem. By developing identification with enterprises characterized as credible, trustworthy, and honest, customers are inclined to depict similar profiles toward themselves (Keh and Xie, 2009; Martínez and Bosque, 2013). Ho (2014) proposes that individuals who trust a brand are likely to identify as a part of the brand community. Martíner and Ignacio (2015) similarly argue that customer identification with an enterprise in the absence of trust is almost impossible. Hence, this study posits that:

H8. Customer trust positively influences customer identification.

On the basis of the above discussions, this study argues that customer trust and identification sequentially mediate the positive effect of ECI quality on customers’ prohibitive voice behaviors. Specifically, ECI quality can establish customers trust (Arnold et al., 2014; Park and Jang, 2014). Customers generally have a good evaluation of enterprises they trust, on which basis they easily develop identification (Martínez and Bosque, 2013; Martíner and Ignacio, 2015). Customer identification in promoting customers’ engagement and extra-role behaviors (Wu et al., 2017; Hur et al., 2018; Roy et al., 2018). Overall, if customers experience high-quality ECI in the consumption process, then they are inclined to trust the enterprises (H2); such trust can improve customer identification with enterprises (H8); and such identification further promotes customers’ prohibitive voice behaviors (H6). As such, ECI quality can positively influence customer’ prohibitive voice behaviors though the sequential mediation of customer trust and identification. Thus, this study hypothesizes that:

H9. Customer trust and identification play sequential mediating roles in the positive effect of ECI quality on customers’ prohibitive voice behaviors.

**FIGURE 1:**
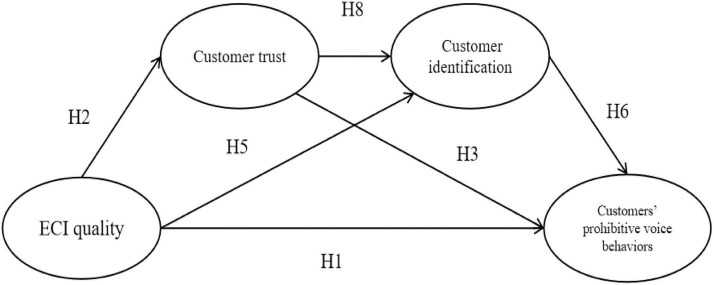
Research model. Mediating paths (H4, H7, and H9) are not presented.

Figure 1 outlines the theoretical model where ECI quality is the antecedent, customer trust and identification are the mediators, and customers’ prohibitive voice behaviors are the outcomes. Customer trust and identification are hypothesized to directly and sequentially mediate the effect of ECI quality on customers’ prohibitive voice behaviors.

## Methodology

### Measurement

Multiple-item 5-point Likert scales ranging from 1 (Strongly Disagree) to 5 (Strongly Agree) were applied to measure all constructs. ECI quality was measured with three items obtained from Lin and Wong (2020). Customer trust was assessed with a three-item scale drawn from Morgan and Hunt (1994). Customer identification was measured with four items provided by Yang et al. (2017). Customers’ prohibitive voice behaviors were evaluated using a three-item scale adapted from Ran and Zhou (2020). Translation and back-translation procedures were administrated to translate the English scales into Chinese. In addition, a panel of researchers and customers in the pre-test helped improve the item statements to ensure suitability in the study context prior to the main survey.

### Data Collection

The target population was defined as customers who had visited a restaurant in the last half month at the time of the survey. The participants were required to be 18 of age or older who were willing to help complete an online survey. The questionnaire was created and distributed *via* social network platforms using a snowball sampling approach in July and August 2020.

COVID–19 had been generally under control in China when the survey was conducted, and more and more people dined out at restaurants. However, to reduce unnecessary interpersonal communications with data collectors, most customers still rejected to help fill in questionnaires in a field survey. Therefore, an online survey presented an alternative way to effectively collect data. In addition, online survey is extensively applied in tourism and hospitality research and is thus suitable for this study (Roy et al., 2018; Wang and Lang, 2019; Taylor, 2020).

Similar to existing research (Yoon and Kim, 2016; Moon et al., 2017), this study used the popular snowball sampling approach which presents advantages of easily reaching large numbers of potential participants with a broad spectrum of sample distribution (Yoon and Kim, 2016). Despite its shortcoming of sample representativeness, snowball sampling approach is an adequate alternative as the use of social networks can increase the sample size and representativeness (Baltar and Brunet, 2012).

Four authors and five assistants contacted their friends and relatives to complete the questionnaire. In turn, the invited participants used their social networks to ask other respondents to fill in the questionnaire. Finally, 395 usable responses were obtained from a pool of 526 returned questionnaires. The final sample is more than 15 times the number of scale items and can thus be considered fairly sufficient (Stevens, 1996).

### Data Analysis

The reliability and validity of all scales were assessed on the basis of results of confirmatory factor analysis (CFA). Then, the proposed hypotheses (H1, H2, H3, H5, H6, and H8) were statistically examined using structural equation modeling (SEM). Finally, the Bootstrapping approach was applied to test the hypotheses for mediation hypotheses (H4, H7, and H9). SPSS 24 and Amos 24 were used for the data analysis in this study.

## Results

### Sample Profiles

Descriptive analysis of sample profiles in Table 1 indicates that 41.5% were male customers and 58.5% were female. The majority of respondents were 25–34 years old (32.9%) followed by those aged 35–44 (27.4%). Most of the respondents held bachelor’s degrees (47.6%). With regard to monthly income, the distribution was 33.2% in the range of CNY 3,001–5,000. The respondents who visited a restaurant for the first time accounted for 49.6%.

**TABLE 1:** Sample profiles.

Profiles	Categories	Number	Percent (%)
Gender	Male	164	41.5
	Female	231	58.5
Age	18–24	76	19.2
	25–34	130	32.9
	35–44	108	27.4
	45–54	69	17.5
	More than 55	12	3.0
Education level	Primary school or below	4	1.0
	Junior high school	27	6.8
	Senior high/Technical secondary school	37	9.4
	Junior college	64	16.2
	Undergraduate	188	47.6
	Master’s or above	75	19.0
Monthly income	CNY 3,000 or less	92	23.3
	CNY 3,001–5,000	131	33.2
	CNY 5,001–7,000	92	23.3
	CNY 7,001–9,000	25	6.3
	CNY 9,001 or more	55	13.9
Frequency of visit	First time	196	49.6
	More than one time	199	50.4

### Common Method Bias

In this study, a cross-sectional design was applied to collect data *via* self-reported questionnaires. Therefore, Common Method Bias (CMB) may occur. This potential issue was addressed using procedural and statistical methods. Procedurally, at the beginning of the questionnaires, participants were informed of their anonymity and confidentiality, and that each question had no right or wrong answer. Statistically, a common factor analysis test was applied to detect the possibility of CMB (Kamboj, 2020). The results indicated that the model fit indices of the simple factor model (2/df = 12.845, GFI = 0.715, CFI = 0.753, TLI = 0.704, RMR = 0.069, and RMSEA = 0.173) were worse than those of the measurement model with four-factors (2/df = 1.506, GFI = 0.967, CFI = 0.990, TLI = 0.987, RMR = 0.018, and RMSEA = 0.036). These results showed that CMB was not a concern in this study.

### Measurement Model

Data normality was assessed using the skewness and kurtosis of each item. The results indicated that the distribution of skewness (ranging from −0.643 to −0.316) and that of kurtosis (from −0.215 to 1.384) were acceptable as suggested by Kline (2011). Thus, the collected data generally accorded with normality assumptions.

Table 2 presents the results of CFA. Cronbach’s alpha (varying from 0.814 to 0.887, greater than 0.60) and composite reliability (ranging from 0.814 to 0.889, larger than 0.60) indicated satisfactory reliability (Fornell and Larcher, 1981; Kline, 2011). In addition, the results of standard factor loadings (varying from 0.751 to 0.878, greater than 0.60) and of average variance extracted (AVE) (ranging from 0.594 to 0.709, larger than 0.50) supported acceptable convergent validity (Fornell and Larcher, 1981).

**TABLE 2:** Results of CFA.

Constructs and items	Means	Standard deviations	Factor loadings	AVE	Composite reliability	Cronbach’s alpha
**ECI quality**				0.594	0.814	0.814
I interact well with employees of this restaurant	3.64	0.840	0.751			
I enjoy interacting with employees of this restaurant	3.64	0.871	0.784			
Interacting with employees of this restaurant makes me feel comfortable	3.78	0.869	0.776			
**Customer trust**				0.709	0.879	0.878
I think this restaurant is reliable	3.95	0.699	0.792			
I have confidence in this restaurant	3.81	0.767	0.878			
I think this restaurant has high integrity	3.87	0.783	0.853			
**Customer identification**				0.667	0.889	0.887
I fairly identify with this restaurant	3.85	0.689	0.817			
I feel good to be a customer of this restaurant	3.81	0.748	0.839			
I like to tell that I am a customer of this restaurant	3.69	0.818	0.825			
This restaurant fits me well	3.70	0.813	0.784			
**Customers’ prohibitive voice behaviors**				0.704	0.877	0.875
I would reflect the possible problems in product and service to the restaurant to help them improve	3.49	0.979	0.864			
I would report the actual problems encountered in receiving service to the restaurant to help avoid its re-occurrence	3.60	0.906	0.826			
I would comment on the issues that are not conducive to the development of the restaurant to improve its performance	3.37	0.950	0.827			

Discriminant validity was assessed using comparisons between the square roots of AVE and the corresponding Pearson correlation coefficients of any pair of constructs. Table 3 shows that the corresponding Pearson correlation coefficients were lower than the square roots of AVE of the concerned constructs, indicating good discriminant validity (Fornell and Larcher, 1981).

**TABLE 3:** Results of discriminant validity test.

Variables	1	2	3	4
1. ECI quality	**0.770**			
2. Customer trust	0.522^**^	**0.842**		
3. Customer identification	0.610^**^	0.808^**^	**0.817**	
4. Customers’ prohibitive voice behaviors	0.566^**^	0.503^**^	0.610^**^	**0.839**

**P < 0.01.

### Structural Model

**FIGURE 2:**
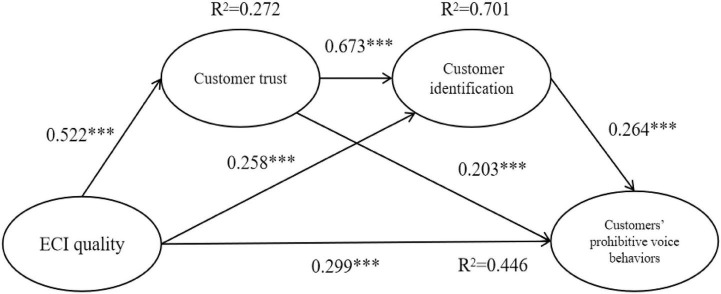
Results of hypothesis test. ***p < 0.001, **p < 0.01, and *p < 0.05.

Figure 2 presents the results of SEM with a satisfied model fit (2/df = 1.506, GFI = 0.967, CFI = 0.990, TLI = 0.987, RMR = 0.018, and RMSEA = 0.036). The structural model explained 27.2%, 70.1%, and 44.6% variance of customer trust, identification, and prohibitive voice behaviors, respectively.

Figure 2 indicates that ECI quality was positively associated with customers’ prohibitive voice behaviors (β = 0.229, *p* < 0.001), trust (β = 0.522, *p* < 0.001), and identification (β = 0.258, *p* < 0.001). Therefore, H1, H2, and H5 were statistically supported. These findings implied that enjoyable interactions with restaurant employees motivated customers to exhibit prohibitive voice behaviors to help the restaurant achieve success. In addition, these findings also indicated that high-quality ECI was a significant accelerator for customer trust toward and identification with the restaurant. Customer trust positively influenced customers’ prohibitive voice behaviors (β = 0.203, *p* < 0.05), and thus statistically supported H3. These findings implied that if customers perceived the restaurant to be reliable and trustworthy, then they tended to benefit the restaurant by offering prohibitive voice behaviors. Customer identification was positively associated with customers’ prohibitive voice behaviors (β = 0.264, *p* < 0.01), and thus statistically supported H6. These findings implied that if customers identified with the restaurant, then they were inclined to exhibit their prohibitive “voices” to give beneficial feedback. Customer trust positively influenced customer identification (β = 0.673, *p* < 0.001), and thus statistically supported H8. These findings implied that customers tended to develop identification with restaurants with high integrity and reliability.

### Mediation Analysis

The Bootstrapping approach with 5,000 samples and 95% confidence intervals was applied to examine the mediating roles of customer trust and identification in the relationship between ECI quality and customers’ prohibitive voice behaviors. Jose (2013) suggested that the mediating effect exists if the confidence interval excludes zero, and none otherwise. Table 4 shows the results of the mediation analysis. The indirect effect of ECI quality on customers’ prohibitive voice behaviors *via* customer trust was 0.106 with a 95% confidence interval [0.008, 0.232]. Therefore, H4 was supported. These results indicated that customer trust played a direct mediating role in the linkage between ECI quality and customers’ prohibitive voice behaviors. Similarly, the indirect effect of ECI quality on customers’ prohibitive voice behaviors *via* customer identification was 0.068 with a 95% confidence interval [0.009, 0.143]. Accordingly, H7 was supported. These results implied that customer identification also played a direct mediating role in the association between ECI quality and customers’ prohibitive voice behaviors. Furthermore, the indirect effect of ECI quality on customers’ prohibitive voice behaviors *via* customer trust and identification was 0.093 with a 95% confidence interval [0.002, 0.187]. These results indicated that customer trust and identification sequentially mediated the effect of ECI quality on customers’ prohibitive voice behaviors. Therefore, H9 was supported.

**TABLE 4:** Results of mediation analysis.

Paths	Indirect effects	Standard errors	Confidence intervals
ECI quality → Customer trust → Customer prohibitive voice behaviors	0.106	0.057	[0.008, 0.232]
ECI quality → Customer identification → Customer prohibitive voice behaviors	0.068	0.034	[0.009, 0.143]
ECI quality → Customer trust → Customer identification → Customer prohibitive voice behaviors	0.093	0.046	[0.002, 0.187]

## Discussion

### Findings

First, the findings support the positive effect of ECI quality on customers’ prohibitive voice behaviors. High-quality ECI is characterized as customers receiving personal care, useful help, and sense of genuineness from employees. The customer–employee interaction can be regarded as a type of social exchange relationship. According to the reciprocity principle in social exchange theory, if customers gain emotion care or useful help in ECI, then they are inclined to give a positive act for the employees or the enterprises as rewards in the social exchange relationship. These findings reconfirm the importance of employee–customer interaction quality in affecting customer attitudes and behaviors in the hospitality industry (Huang and Xie, 2017; Wang and Lang, 2019). The results also support those of Yi and Gong (2006) that high-quality ECI is an important accelerator of customers’ extra-role behaviors. This finding expands research conclusions of Yang and Hao (2020) from the enerprise-customer perspective and discovers another antecedent variable that promotes customer voices from the employee-customer perspective.

In addition, the present findings indicate that customer trust and identification not only directly but also sequentially mediate the positive effect of ECI quality on customers’ prohibitive voice behaviors. If customers gain personal care and attention from employees in service interactions, then they tend to develop favorable evaluations toward employees and thereby establish trust-based relationships with service enterprises. In addition, many existing studies provide statistical evidence for the role of ECI quality in driving customer trust. Based on the social exchange theory and the norm of reciprocity, the senses, and experience of customers interacting with service providers can generate emotional trust. Customer trust is expected to promote the supportive extra-role behavior of customers toward the enterprise. According to the social identity theory, strong sense of common identity makes individuals see the organization as part of themselves. Customers are “partial employees” of the company, and their trust and sense of responsibility in the company will promote their opinions and behaviors. Therefore, customers with a high level of identification will be more motivated to show voluntary and positive behavior toward the company. The findings imply that when customers have enjoyable interactions with employees, they are inclined to trust and identify with the restaurant. In turn, such trust and identification simply and jointly promote customer behaviors of expressing prohibitive voices toward the restaurant.

Finally, the direct mediating roles of customer trust and identification in this study provide further evidence of their mediation effects in external stimuli on customer responses (Keh and Xie, 2009; Martínez and Bosque, 2013). The sequential mediating roles further open the “black boxes” of how ECI quality affects customers’ prohibitive voice behaviors, and support the existence of the indirect path of “ECI quality → Customer trust → Customer identification → Customers’ prohibitive voice behaviors”. If customers enjoy high ECI quality during the consumption process, they are more inclined to trust the company; this trust can increase the customer’s identification of the company. This identification further promotes customer’s prohibitive voices. Therefore, ECI quality have a positive impact on customers’ prohibitive voice behavior through the intermediary role of customer trust and identification. Such findings highly improve the understanding of the underlying mechanism of the effect of ECI quality on customers’ prohibitive voice behaviors from the perspective of relational benefit enhancement of customers.

### Theoretical Contribution

First, the differences between employee voice and complaint behaviors have been explicitly addressed, this study is a pioneer attempt to thoroughly differentiate the concept of customer voice behaviors from that of customer complaint behaviors. Specifically, customer voice behaviors are committed to benefiting enterprises and can occur in the absence of discontent, which essentially pertain to customer citizenship behaviors. Meanwhile, customer complaint behaviors aim to express customers’ dissatisfaction and negative emotions from unpleasant experiences or service failure in the consumption process, which do not pertain to customer citizenship behaviors. These important distinctions can provide useful reference value for future research on related topics. This study draws on the research of Liang et al. (2012) and Ran and Zhou (2020) about the classification of employee voices. We divide customer voices into promotive and prohibitive voices, which fill the gap in customer voice behavior. The new definition and dimension of customer voice proposed in this paper will help existing research on customer voice behavior and provide inspiration for future researchers.

Second, this study responds to Ran and Zhou (2020) call to pay attention to customer voice behaviors. Customer citizenship behaviors are extensively addressed in past literature (Groth, 2005; Hur et al., 2018; Kim et al., 2018), but limited research focuses on customer voice behaviors, especially on the prohibitive ones. This study focuses customers’ prohibitive voice behaviors and enriches the existing literature on the predictors of such behaviors from the perspective of service interactions. The findings support the driving effect of ECI quality on customers’ prohibitive voice behaviors. To the best of the authors’ knowledge, this study is the first to empirically investigate the drivers of customer voice behaviors by addressing the roles of employees, and thereby helps deepen the understanding of such roles in the formation of customer citizenship behaviors. Existing research explores the impact of customer – corporate identification (Yang and Hao, 2020) on customer’s voice behavior, while this article explores more remote factors and verifies the positive behavior of customers on service contact and after purchase by Zolfagharian et al. (2018). This paper finds that ECI quality has a positive impact on customers’ prohibitive voice behaviors. This study presents a pioneer attempt to explore the role of ECI quality in predicting customers’ prohibitive voice behaviors. This study is a valuable addition to the antecedents of such behaviors from the perspective of service interactions and provides helpful practical implications for its stimulation.

Third, the results of this paper show that customer trust and identification have a positive impact on customers’ forbidden voices. This validates the work of Roy et al. (2018) and Wang and Lang (2019) and expands their application areas. The results of this study extend the research of Wang and Lang (2019) and find that the quality of customer-employee interaction can improve the prohibited behaviors through customer identification, not just promotive voices. This research verifies the positive effects of Grissemann and Stokburger-Sauer (2012) and Ran and Zhou (2020) on the role of customers as “partial employees.”

Finally, this study analyzes the underlying mechanism of how ECI quality indirectly affects customers’ prohibitive voice behaviors by examining the mediating roles of customer trust and identification. The mediating roles of customer trust and identification in the relationships between customer perceptions and behaviors have been widely explored (Ahearne et al., 2005; Martínez and Bosque, 2013; Kim et al., 2018), but scant research applies these two constructs to link customers’ perceptions of employee-related factors and customers’ extra-role behaviors. The present study adds evidence to current knowledge by exploring the direct and sequential mediating roles of customer trust and identification, and provides a comprehensive understanding of the effects of these two constructs on customers’ extra-role behaviors. To stimulate customer voice behaviors, enhancing customer trust and identification is necessary.

This article integrates two relationship variables (customer trust and recognition), and opens up the “black box” of how ECI quality indirectly affects the forbidden speech behavior of customers by establishing relationship benefits. This integration greatly improves the work of Ran and Zhou (2019, 2020), which has not yet focused on upstream predictive factors for customer identification. The research in this article finds that customer trust and identification play sequential mediating roles in the positive effect of ECI quality on customers’ prohibitive voice behaviors. This result further rationalizes the logical relationship between employee-customer relationship quality, customer trust, customer recognition and customer voice behaviors.

### Practical Implication

The findings indicate that ECI quality has positive effects on customer trust, identification, and prohibitive voice behaviors. Therefore, effectively improving ECI quality in the restaurant context is important. Specifically, restaurant managers are suggested to pay considerable attention to improving employees’ communicative skills through targeted training. Important procedures characterized by frequent ECI, such as inquiry, ordering, and settling accounts, should be highlighted. Employees’ willingness to interact with customers is a basis of high-quality ECI. Accordingly, restaurant managers can implement measures to encourage employees to sincerely interact with customers, attach importance to customers’ personal preference, and provide customers with useful help. An important consideration is that employees should protect the personal privacy of customers in the ECI to prevent the latter from feeling upset and worried. Alternatively, an incentive system regarding ECI quality can be formulated on the basis of customer comments and scores.

In addition to the above measures for improving ECI quality, restaurant managers can use other means to promote customer trust and identification, which are found to play significant roles in predicting customers’ prohibitive voice behaviors. For example, restaurant managers can foster customer trust and identification by improving service quality, enhancing firm reputation, reinforcing consumer participation, and strengthening corporate social responsibility (Keh and Xie, 2009; Martínez and Bosque, 2013; Ho, 2014). Thus, the goal of further promoting customers’ prohibitive voice behaviors can be achieved.

For another example, before the COVID-19 pandemic, the interaction between employees and customers was more face-to-face, and employees can increase service content and targeted recommendations for customers, which helps customers make appropriate choices and enhance customer trust and identification. During the epidemic, companies actively implemented social responsibilities and ensured food safety. Employees take protective measures, and provide customers with free masks, disinfection water. And employees should remind customers to pay attention to safety protection. These ECI promote customer trust and identification and encourage consumer voice behaviors. After the epidemic, more discounts will be given to customers who have made contributions to the epidemic to encourage their contributions. These can not only gain customers’ trust and identification, but also further encourage them to participate in epidemic prevention and control activities. Customers can gain recognition and trust, and encourage them to give more voices *via* increasing the interaction between employees and customers.

## Conclusion

On the basis of social exchange and identity theories, this study explores the effect of ECI quality on customers’ prohibitive voice behaviors in the restaurant context. The mediating roles of customer trust and identification are particularly examined. Results of this study show that ECI quality positively affects customers’ prohibitive voice behaviors. In this effect, customer trust and identification play direct and sequential mediating roles. This result further rationalizes the logical relationship between employee-customer relationship quality, customer trust, customer recognition and customer voice behaviors. This study contributes theoretically to the current knowledge by clearly distinguishing customer voice behaviors from customer complaint behaviors and by providing new insights into the mechanism of customers’ prohibitive voice behaviors from the perspectives of service interaction and relational benefit enhancement. The practical implications of this study can help pointedly foster customers’ prohibitive voice behaviors.

This study acknowledges a few limitations. First, this article explores the impact of customer-employee interaction quality on customer voice behaviors. However, the customer’s voice behaviors may be affected by factors such as the customer’s own characteristics and corporate behaviors. Therefore, we encourage more research on the potential mechanism of customer voice behaviors and the results of customer voice behaviors from different theoretical perspectives. Second, the non-probability sampling technique in the online survey may lead to concerns on sample representativeness. Hence, other sampling methods can be applied for data collection to further examine the findings. The self-reported survey in a cross-sectional design may cause response biases and has limited persuasiveness to illustrate causal relationships among the variables. Future research is therefore encouraged to employ multi-source survey data based on longitudinal designs to re-examine the proposed hypotheses. Third, another limitation is that this study was conducted merely in the restaurant context in China, thus, the findings may have limited generalization in other contexts. Future research can replicate the model in other service contexts and diversely cultural settings. Finally, this study was only done in high contact industries, whether these findings apply to low contact industries remains open to debate. Thus, future research can be conducted similar research in the low contact industries context.

## Data Availability Statement

The original contributions presented in the study are included in the article/supplementary material, further inquiries can be directed to the corresponding author.

## Author Contributions

GC and SL: conceptualization, methodology, software, and validation. SL: formal analysis, investigation, resources, data curation, and writing—original draft preparation. GC: writing—review and editing, visualization, supervision, and project administration. Both authors contributed to the article and approved the submitted version.

## Conflict of Interest

The authors declare that the research was conducted in the absence of any commercial or financial relationships that could be construed as a potential conflict of interest.

## Publisher’s Note

All claims expressed in this article are solely those of the authors and do not necessarily represent those of their affiliated organizations, or those of the publisher, the editors and the reviewers. Any product that may be evaluated in this article, or claim that may be made by its manufacturer, is not guaranteed or endorsed by the publisher.
